# Only multi-taxon studies show the full range of arthropod responses to fire

**DOI:** 10.1371/journal.pone.0195414

**Published:** 2018-04-03

**Authors:** Inam Yekwayo, James S. Pryke, René Gaigher, Michael J. Samways

**Affiliations:** 1 School of Biology and Environmental Sciences, University of Mpumalanga, Nelspruit, South Africa; 2 Department of Conservation Ecology and Entomology, Stellenbosch University, Matieland, South Africa; University of Roehampton, UNITED KINGDOM

## Abstract

Fire is a major driver in many ecosystems. Yet, little is known about how different ground-living arthropods survive fire. Using three sampling methods, and time-since-fire (last fire event: 3 months, 1 year, and 7 years), we investigate how ground-living arthropod diversity responds to fire, and how species richness, diversity, abundance, and composition of the four dominant taxa: ants, beetles, cockroaches and mites, respond. We did this in the naturally fire-prone Mediterranean-type scrubland vegetation (fynbos) of the Cape Floristic Region. Surprisingly, overall species richness and diversity was the same for all time-since-fire categories. However, when each dominant taxon was analysed separately, effect of fire on species richness and abundance varied among taxa. This emphasizes that many taxa must be investigated to really understand fire-driven events. We also highlight the importance of using different diversity measures, as fire did not influence species richness and abundance of particular taxa, while it affected others, overall greatly affecting assemblages of all taxa. Rockiness affected species richness, abundance and composition of a few taxa. We found that all time-since-fire categories supported distinctive assemblages. Some indicator species occurred across all time-since-fire categories, while others were restricted to a single time-since-fire category, showing that there is a wide range of responses to fire between taxa. Details of local landscape structure, abiotic and biotic, and frequency and intensity of fire add complexity to the fire-arthropod interaction. Overall, we show that the relationship between fire and arthropods is phylogenetically constrained, having been honed by many millennia of fire events, and highly complex. Present-day species manifest a variety of adaptations for surviving the great natural selective force of fire.

## Introduction

The importance and the effect of fire on plants is fairly well known [1]. Yet its effect on arthropods is poorly understood [2]. Arthropods play an important functional role in many ecosystem processes, such as nutrient cycling, pollination, decomposition, and food web interactions [3,4]. This means that it is important to understand arthropod diversity response and recovery to fire for the long-term maintenance of communities. However, little information is available on arthropod adaptive strategies to fire, especially in the species-rich fynbos.

Fire can have a negative influence on arthropod diversity [5], partly it appears from arthropods, especially insects, having a close relationship with specific plants [6,7]. At the time of a fire, arthropods may flee, or find a refuge to survive the fire [5]. Certain winged species may be able to fly away from the fire, and return after it. However, surviving fires by flying away may require nearby unburned patches for arthropods to inhabit until after the fire, and often until vegetation re-grows. For flightless immature stages and those arthropods that cannot fly, risk of death is high, for example, fire kills many butterfly pupae on the soil surface [8]. Furthermore, it is not well understood how remnant unburned patches are affected by fire, especially as they may have much higher abundance of herbivores and other arthropod guilds straight after fire [5]. Resource overuse might explain the observed decrease in arthropod diversity, one year after a major fire event in fynbos [2]. In turn, ground-living arthropods have limited dispersal abilities and are sensitive to environmental disturbances. They rely largely on the limited resources available to them within their restricted habitats [9,10], making fires catastrophic events for them.

For ground-living arthropods that remain in the fire area, they must find refuges, such as in plant stems or roots, underground, or among rocks [8,11,12]. Ground-dwelling ants are able to survive fire by using their underground nests as fire refuges [2,11] as long as the nests are > 10 cm under the soil surface at the time of the fire [5]. In turn, spider species richness and abundance in rocky areas is unaffected by fires because they use rocks and/or plants as shelters from the fire [2]. However, survival depends on the intensity of the fire and its pattern of local movement. Arthropods can also survive if fire intensity is uneven across the landscape, allowing some individuals to survive and re-establish populations.

Here we investigate ground-living arthropod species richness, abundance, and composition responses to fire in fynbos vegetation. This scrubland vegetation type, at the south-western tip of Africa in the Cape Floristic Region, is characterised by high plant diversity, with many highly localized endemic species, and driven by fire [13,14]. We assess arthropod species richness, composition and abundance at three time intervals: 1) 3 months after fire, 2) 1 year after fire, and 3) >7 years after fire. We hypothesise that the three time-since-fire categories will support different arthropod assemblages, and propose that there will be lower arthropod diversity in the 3-month and the 1-year categories compared to the 7-year category. We also assess the effect of micro-topography (rockiness) on arthropod species richness, composition and abundance in the three time-since-fire categories. We expect rock crevices and overhangs to support greater species diversity and different species composition than local areas where no such presumed refuges are available [15].

## Materials and methods

### Study area and site selection

**Fig 1:**
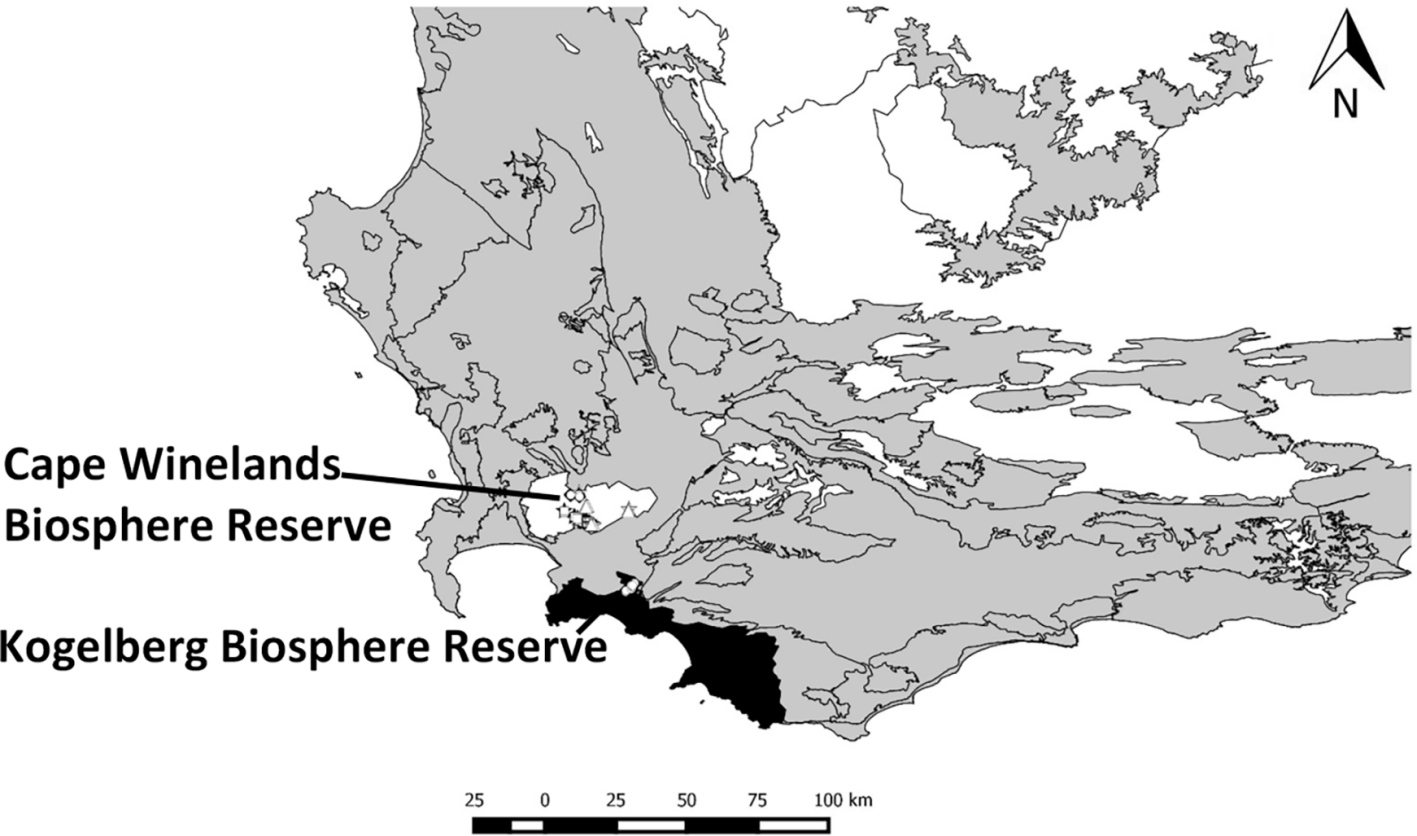
Location of the study sites. Fynbos biome (grey), Cape Winelands Biosphere Reserve (clear), Kogelberg Biosphere Reserve black).

The study was conducted in the Cape Winelands Biosphere Reserve (CWBR) (33°56'46.57"S, 19°7'50.16"E) and Kogelberg Biosphere Reserve (KBR) (34°15'02.1"S, 019°07'52.9"E), Western Cape, South Africa (Fig 1). The CWBR and KBR are characterized by high mountains with steep slopes with scrubby fynbos vegetation dominated by the plant families Proteaceae, Ericaceae and Restionaceae [16]. These reserves have a Mediterranean climate with warm, dry summers and cool, rainy winters. The fynbos is naturally fire prone and fire is a critical ecosystem process for this biome [17] with recommended burn cycles of 12−15 years [18]. This region is currently experiencing an anthropogenic acceleration in the frequency of fires, due to arson, runaway control burns or recreational fires, discarded cigarettes, etc., and the fires are burning at least twice the natural frequency. In fact, here the oldest age class of fynbos that we could find was 7 years since the previous fire [19].

Our sites were between 236 and 598 m asl, and were in three categories based on time since last fire: 1) 3 months, 2) 1 year, and 3) >7 years. We selected 90 sites, 30 sites for each of the time-since-fire categories. These 30 sites per category, in turn, were divided into 15 rocky and 15 non-rocky sites. However, as 15 non-rocky sites in the >7 year category were unavailable, we used seven non-rocky sites, and 23 rocky sites. The distance between rocky and non-rocky sites within the same category was ≥ 100 m. The distance between sites in the same treatment was ≥ 300 m to avoid pseudoreplication. The size of each study site was 10 x 10 m. Fynbos predominately grows on shallow sandy soils, although there are clay areas, often associated with depressions and wet areas [17]. Here we focused on only the sandy soils with and without rocks.

### Arthropod sampling

Arthropods were sampled using three techniques (pitfall traps, active searching, and suction sampling) to capture a range of taxa. Data from the three techniques were pooled for analyses. Active diurnal searches sampled arthropods that live under stones, suction sampling sampled arthropods living near the base of low fynbos plants (< 1 m tall), while pitfall trapping caught surface-active arthropods [20]. Sampling took place from March−April 2016 (warm and dry late summer).

At each site, four pitfall traps were placed, forming a 2 m^2^ square in each in the corner of the 10 x 10 m site, giving 16 pitfalls per site. Each pitfall trap was a plastic jar 6 cm diameter x 9 cm height, quarter filled with 50% ethylene glycol. Traps were left open for seven days. Data from the 16 traps at each site were pooled for analyses.

At each site, active searching was conducted for 30 min within the 10 x 10 m site. All highly visible ground-living arthropods were collected. Suction sampling made use of a Stihl SH 86 leaf shredder, with a 15 cm diameter nozzle and a bag of fine mesh [21], and effective for fynbos arthropod sampling [22]. Arthropods were sucked from low fynbos plants using 60 insertions in the 10 x 10 m site. Material was stored in bags and frozen, for later sorting in the laboratory.

All specimens were preserved in 70% ethyl alcohol, sorted to morphospecies, and later identified to family, genus, and to species where possible. In hyper diverse areas, and areas with poor taxonomic resolution, the “Linnean shortfall” is a major constraint to arthropod biodiversity studies [23]. To overcome this, the morphospecies approach is often used, which involves creating a reference collections and assigning each individual a pseudonym (or morphospecies) [24]. The reference collection is housed at the Stellenbosch University Entomological Museum and is available for expert identification when available.

### Data analyses

Prior to data analyses, singletons and doubletons were removed, to avoid their influence on the results [25]. Non-parametric species estimators of Chao1, Chao2, Jacknife2 and MM were calculated in PRIMER using 9999 permutations to predict asymptotic species richness for overall data, as well as for each of the four dominant taxa (ants, beetles, cockroaches and mites) [26]. Generalized linear mixed models (GLMMs) were created in R using *lme4* package to test the effect of time-since-fire, rockiness and the interaction between these factors for each of species richness, abundance and inverse Simpson’s diversity index [27]. GLMMs were fitted by Laplace approximation to determine the likelihood estimate for each GLMM parameter, as well as had Poisson error distribution and log-link function [28]. Two models were created. In the first model, time-since-fire and rockiness (rocky vs. non-rocky) were fixed factors, while elevation nested within the sampling area was a random factor. The second model consisted of time-since-fire, rockiness, as well as the interaction between time-since-fire and rockiness as fixed factors (only interaction results are reported from this 2nd order model). Analyses were performed for overall arthropods, as well as for each of the four dominant taxa (ants, beetles, cockroaches, and mites). The Simpson’s diversity and abundance models fitted to a negative binomial distribution, while species richness data fitted to a Poisson distribution [28]. For the Simpson’s diversity, species richness and abundance, analyses showed no over-dispersion of variance for overall arthropods (when pooled) and for each of the four dominant taxa. χ^2^ and p-values were calculated. To perform Tukey post hoc tests on significant factors, the *multicomp* package in R [29] was used.

To test the effect of time-since-fire, rockiness and the interaction between these factors on species composition, we used permutational multivariate analysis of variance (PERMANOVA) in PRIMER 6 (2009, PRIMER-E Ltd). Time-since-fire, rockiness and the interaction between rockiness and time-since-fire were used as fixed factors, and elevation nested within the sampling area was used as a random factor. To reduce the weight of common species, a square-root transformation was used, and analyses were performed using Bray-Curtis similarity measures [30]. F- and p-values were calculated using 9999 permutations [31]. Effect of time-since-fire effect and rockiness on species composition were also determined using canonical analysis of principal coordinates in PRIMER [32].

Arthropods that were indicator species in each category of time-since-fire and those shared among these categories were identified using the indicator value (IndVal) analyses in R [33]. *Indicspecies* package was used [33]. χ^2^ and p-values were provided for significant indicator species (< 0.05). IndVal focuses on the uniqueness of a species to a particular habitat, and its frequency of occurrence in this particular habitat [20,34].

## Results

A total of 27 659 individuals representing 210 morphospecies of arthropods were sampled. Ants contributed highest number of individuals (85.31%), while scorpions were least abundant (0.04%). Overall species richness estimators indicated similar proportions to sampled species richness (observed = 210, Chao1 = 210 ± 3.57, Chao2 = 215.16 ± 3.52, Jacknife2 = 216.36, MM = 231.40). Furthermore, estimated species richness was similar to the observed figure for ants (observed = 73, Chao1 = 73 ± 1.41, Chao2 = 73.89 ± 1.33, Jacknife2 = 72.17, MM = 76.33), beetles (observed = 79, Chao1 = 79 ± 2.35, Chao2 = 81.23 ± 2.35, Jacknife2 = 82.13, MM = 95.53), cockroaches (observed = 17, Chao1 = 17 ± 3.13, Chao2 = 19 ± 3.74, Jacknife2 = 19.97, MM = 21.69), and mites (observed = 27, Chao1 = 27 ± 1.56, Chao2 = 27.9 ± 1.46, Jacknife2 = 28.07, MM = 28.37).

**Fig 2:**
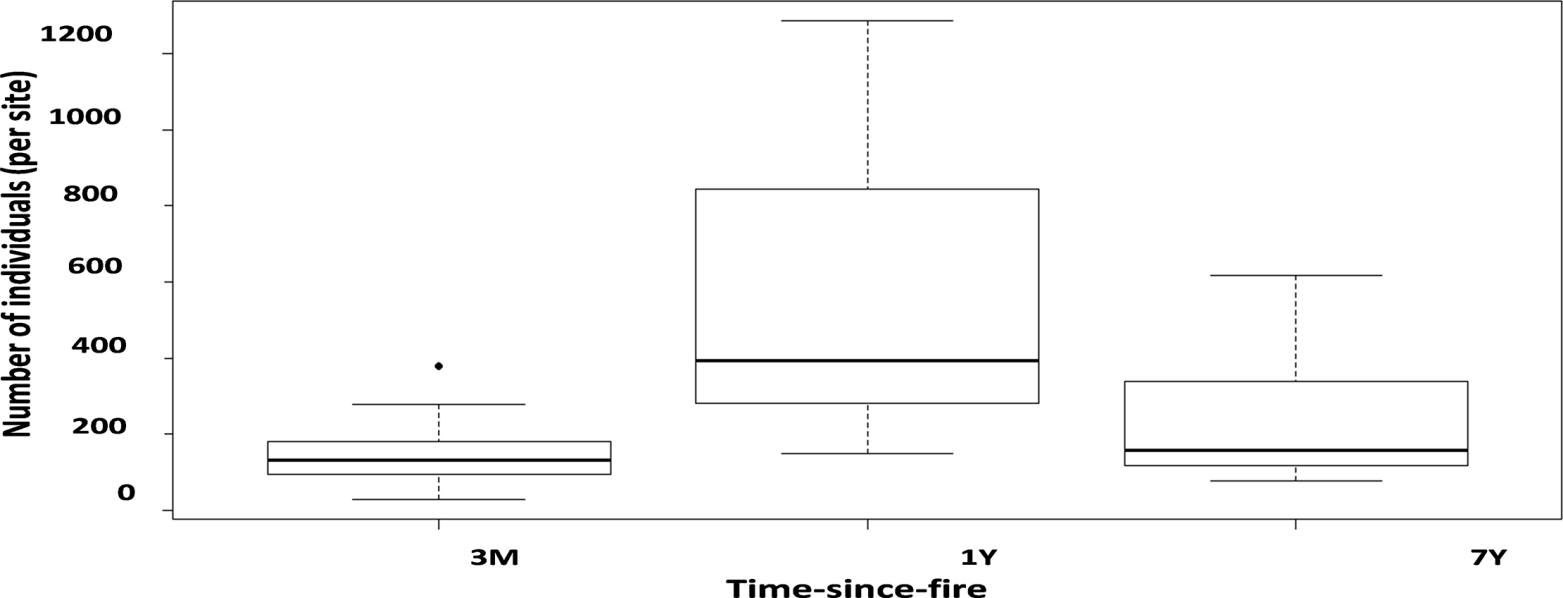
Effect of time-since-fire on the overall arthropod abundance. 3M (3-month category), 1Y (1-year category), 7Y (7-year category).

**Fig 3:**
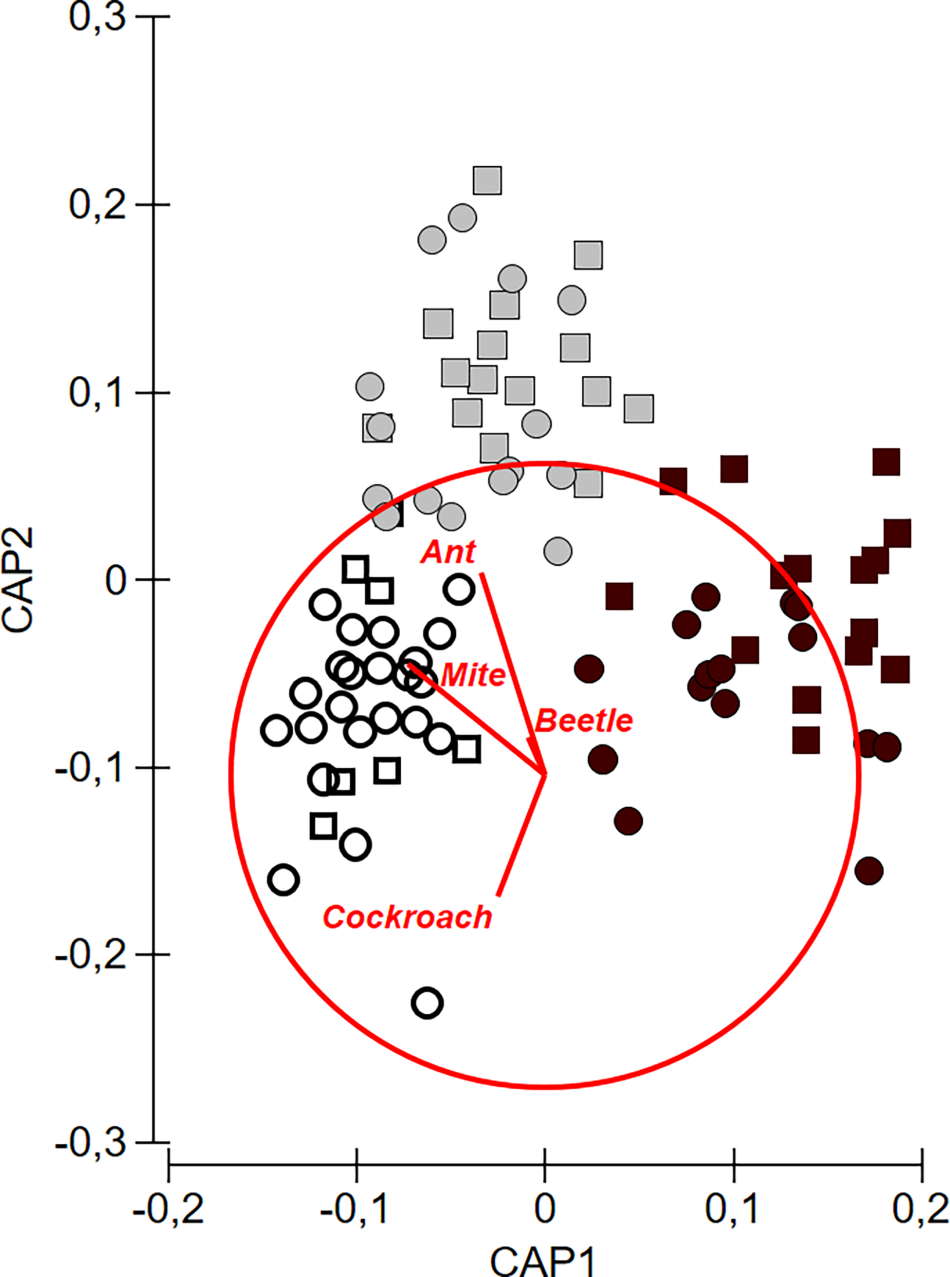
Canonical analysis of principal coordinates indicating effect of rockiness and time-since-fire on overall arthropod species composition. 3-month category (black), 1-year category (grey), 7-year category (clear), circles (rocky), squares (non-rocky).

Simpson’s index and species richness showed that overall arthropod diversity was not influenced by time-since-fire, rockiness or interaction between these factors (Table 1). However, in the case of abundance, time-since-fire was an important factor, with greater abundance in the 1-year category compared with the other two (Table 1; Fig 2). Overall arthropod abundance did not differ between the 7-year and the 3-month categories (Fig 2). However, time-since-fire significantly influenced overall species composition, with distinct dissimilarities in assemblages recorded in each time-since-fire category (Table 1; Fig 3). Overall arthropod species composition was not influenced by the rockiness and the interaction between rockiness and time-since-fire (Table 1; Fig 3).

**Table 1:** Effect of time-since-fire, rockiness and the interaction between these factors, on overall arthropod species richness, abundance, diversity and composition.

	**Species richness**			**Abundance**		
	df	χ^2^	p	df	χ^2^	p
Time-since-fire (TSF)	2	0.67	0.71	2	12.94	< 0.01
Rockiness	1	0.07	0.79	1	0.26	0.61
TSF*Rockiness	2	0.84	0.66	2	0.06	0.96
	**Simpson’s index**			**Species composition**		
	df	χ^2^	p	df	Pseudo-F	p
Time-since-fire	2	0.38	0.83	2	4.20	< 0.001
Rockiness	1	0.02	0.92	1	1.21	0.19
TSF*Rockiness	2	0.05	0.98	2	1.25	0.11

**Fig 4:**
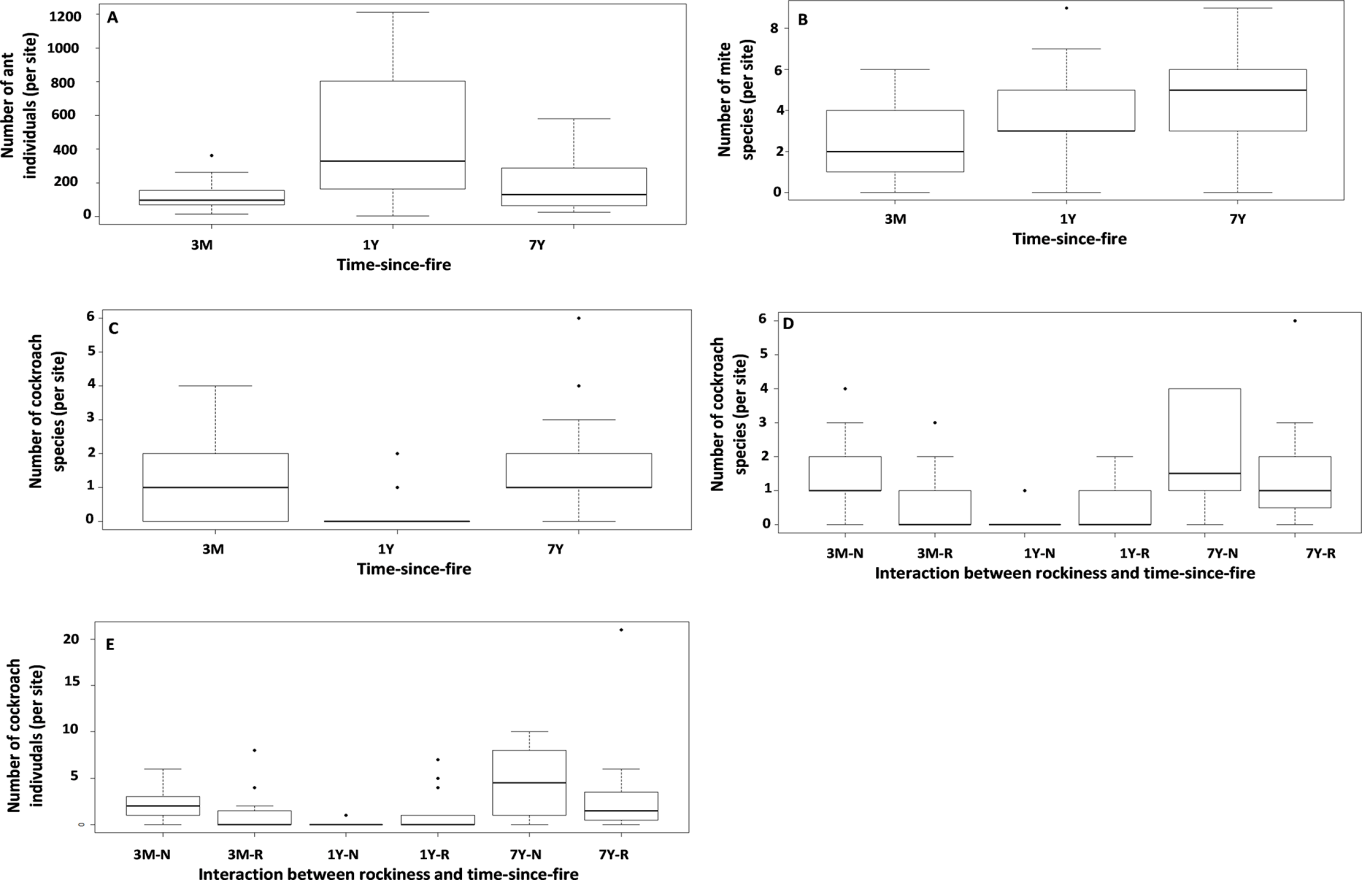
**Effect of time-since-fire on (A) ant abundance, and species richness of (B) mites, (C) cockroaches, effect of the interaction between time-since-fire and rockiness on (D) cockroach species richness and (E) cockroach abundance.** 3M (3-month category), 1Y (1-year category), 7Y (7-year category).

When arthropod taxa were analysed separately, results varied among them. For ants and beetles, time-since-fire, rockiness and interaction between rockiness and time-since-fire were not significant in terms of species richness and diversity (Tables 2 and 3). However, time-since-fire significantly affected abundance of ants and beetles (Table 2). There was higher ant abundance in the 1-year category compared to the other two categories (Fig 4A). Although time-since-fire significantly affected beetle abundance, there were no significant differences among time-since-fire categories (Table 2). Rockiness and the interaction between time-since-fire and rockiness did not affect beetle abundance (Table 2).

**Table 2:** Effect of time-since-fire, rockiness and the interaction between these factors, on species richness (roman type) and abundance (italics and in brackets) of the four dominant arthropod taxa.

	**Ants**			**Beetles**			**Cockroaches**			**Mites**		
	df	χ^2^	p	df	χ^2^	p	df	χ^2^	p	df	χ^2^	p
Time-since-fire (TSF)	2 (*2*)	2.37 (*13*.*29*)	0.30 (*< 0*.*01*)	2 (*2*)	3.25 (*6*.*52*)	0.19 (*< 0*.*05*)	2 (*2*)	10.19 (*4*.*31*)	< 0.01 (*0*.*11*)	2 (*2*)	8.56 (*5*.*23*)	< 0.05 (*0*.*07*)
Rockiness	1 (*1*)	1.24 (*0*.*26*)	0.26 (*0*.*61*)	1 (*1*)	1.57 (*0*.*00*)	0.21 (*0*.*99*)	19 (*1*)	1.98 (*0*.*00*)	0.16 (*0*.*95*)	1 (*1*)	2.95 (*1*.*29*)	0.08 (*0*.*26*)
TSF*Rockiness	2 (*2*)	1.84 (*0*.*10*)	0.39 (*0*.*95*)	2 (*2*)	1.42 (*1*.*56*)	0.49 (0.46)	2 (*2*)	6.86 (*10*.*32*)	< 0.05 (< *0*.*01)*	2 (*2*)	0.52 (*1*.*52*)	0.77 (*0*.*47*)
**Pairwise comparison of abundance and species richness among time-since-fire categories. Only significant posthoc results are shown.**												
	**Abundance**						**Species richness**					
	**Ants**			**Beetles**			**Cockroaches**			**Mites**		
	z	p		z	p		z	p		z	p	
3M – 1Y	(*-3*.*97*)	(*< 0*.*001*)		(*-2*.*17*)	(*0*.*07*)		2.04	0.10		-2.65	< 0.05	
3M – 7Y	(*-0*.*70*)	(*0*.*76*)		(*-0*.*05*)	(*0*.*99*)		-1.42	0.33		-3.28	< 0.01	
7Y – 1Y	(*-3*.*37*)	(*< 0*.*01*)		(*-2*.*08*)	(*0*.*09*)		3.38	< 0.01		0.55	0.85	

3M (3-month category), 1Y (1-year category), 7Y (7-year category).

**Table 3:** Effect of time-since-fire, rockiness and the interaction between these factors, on composition of assemblages (roman type) and on Simpson’s diversity (italics and in brackets) of the four dominant arthropod taxa.

	**Ants**			**Beetles**			**Cockroaches**			**Mites**		
	df	Pseudo-F (*χ*^2^)	p	df	Pseudo-F (*χ*^2^)	p	df	Pseudo-F (*χ*^2^)	p	df	Pseudo-F (*χ*^2^)	p
Time-since-fire	2 (*2*)	3.06 (*0*.*61*)	< 0.001 (*0*.*74*)	2 (*2*)	3.49 (*0*.*37*)	< 0.001 (*0*.*83*)	2 (*2*)	4.54 (*8*.*00*)	< 0.001 (*< 0*.*05*)	2 (*2*)	5.08 (*2*.*39*)	< 0.001 (*0*.*30*)
Rockiness	1 (*1*)	1.26 (*0*.*01*)	0.20 (*0*.*93*)	1 (*1*)	1.22 (*0*.*19*)	0.22 (*0*.*66*)	1 (*1*)	0.95 (*0*.*81*)	0.44 (*0*.*37*)	1 (*1*)	2.95 (*8*.*36*)	0.85 (*< 0*.*01*)
TSF*Rockiness	2 (*2*)	1.04 (*0*.*62*)	0.39 (*0*.*97*)	2 (*2*)	1.42 (*0*.*03*)	< 0.05 (*0*.*66*)	2 (*2*)	1.31 (*1*.*74*)	0.20 (*0*.*42*)	2 (*2*)	0.86 (*35*.*45*)	0.64 (*< 0*.*001*)
**Pairwise comparison of assemblage composition among time-since-fire categories.**												
	**Ants**			**Beetles**			**Cockroaches**			**Mites**		
	t	p		t	p		t	p		t	p	
7Y – 1Y	1.87	< 0.001		1.52	< 0.001		1.41	0.06		2.44	< 0.001	
3M – 1Y	1.69	< 0.001		2.01	< 0.001		2.24	< 0.01		2.29	< 0.001	
3M – 7Y	1.71	< 0.001		2.01	< 0.001		2.46	< 0.001		2.04	< 0.001	

3M (3-month category), 1Y (1-year category), 7Y (7-year category).

Mite abundance and diversity were not influenced by time-since-fire (Tables 2 and 3). However, there was significantly higher mite species richness in the 7-year and 1-year categories compared to the 3-month one (Table 2; Fig 4B). Species richness of mites in the 7-year category did not differ from that in the 1-year category (Fig 4B). In turn, mite diversity was significantly influenced by rockiness and the interaction between rockiness and time-since-fire (Table 3). However, there were no differences in mite species diversity or species richness or abundance in rocky vs. non-rocky sites or any paired interactions (Tables 2 and 3). Higher diversity and species richness of cockroaches was recorded in the 7-year category compared to the 1-year category (Tables 2 and 3; Fig 4C). In turn, cockroach species richness in the 3-month category did not differ from the 7-year or the 1-year categories (Table 2; Fig 4C). Cockroach species richness was not affected by rockiness (Table 2). Similarly, there were similarities in abundance of cockroaches among time-since-fire categories, as well as between rocky vs. non-rocky sites (Table 2). However, interaction between rockiness and time-since-fire affected species richness and abundance of cockroaches, with greater abundance and richness in the 7-year category (rock and non-rocky) than the 1-year non-rocky sites (Table 2; Fig 4D and 4E).

**Fig 5:**
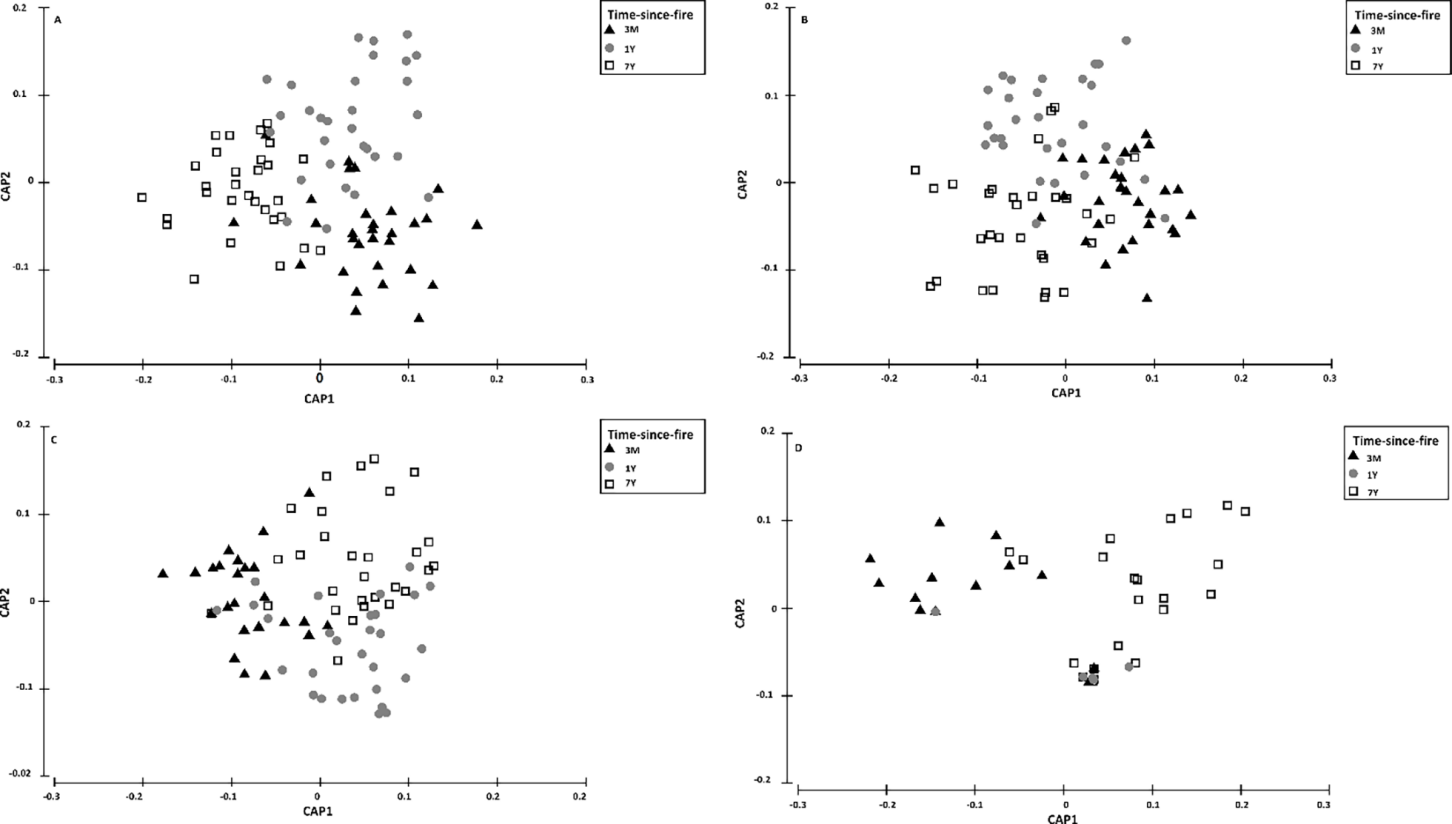
**Canonical analysis of principal coordinates (CAP) of (A) ants, (B) beetles, (C) mites, and (D) cockroaches across time-since-fire categories.** 3M (3-month category), 1Y (1-year category), 7Y (7-year category).

Ant species composition was significantly influenced by the time-since-fire, with all three time-since-fire categories supporting different assemblages (Fig 5A). Although not significant, there were overlaps of ant assemblages among time-since-fire categories (Fig 5A). Species composition of ants, beetles, cockroaches and mites did not differ between rocky vs. non-rocky sites (Table 3). However, for beetles, interaction between time-since-fire and rockiness significantly influenced assemblage composition (Table 3). Most categories supported different beetle species composition, with the exception of the 7-year rocky and 7-year non-rocky sites, 1-year rocky and 7-year rocky sites, and 1-year rocky and 1-year non-rocky sites (Table 3). All three categories of time-since-fire supported significantly different assemblages of beetles and mites, although there were some overlaps in assemblages (Fig 5B and 5C). There were also similarities in species composition of cockroaches in the 7-year and 1-year categories, while the 3-month category differed from both the 1-year and 7-year categories (Fig 5D).

Indicator value (IndVal) analyses identified five species (one millipede, one ant, one cockroach, and two beetles) as indicators of the 3-month category (S1 Table). The 1-year category had one ant, one beetle, and one mite species as indicators (S1 Table). There were 15 indicator species of the 7-year category; one pseudoscorpion, one millipede, two mites, two ants, four cockroaches, and five beetles (S1 Table).

The 3-month category shared four ant species with the 1-year category, while there were no indicator species shared between the 3-month and 7-year categories (S1 Table). Three beetle, three mite, and one cockroach species were shared indicators between the 1-year and 7-year categories (S1 Table). There were three species (two *Camponotus* sp. and a Harpagophoridae sp.) that occurred frequently in all three time-since-fire categories (S1 Table).

## Discussion

We demonstrate here that time-since-fire does not influence overall arthropod species richness or overall species diversity. However, when each major taxon was analysed separately, the results showed that the influence of fire on species richness, as well as on abundance, vary greatly among taxa. Contrary to our expectations, degree of rockiness, i.e. micro-topography, of the habitat did not affect the overall arthropod assemblages. This was surprising as we expected rocky sites to provide refuges through crevices, shelter beneath and in-between rocks, indicating that rock crevices and overhangs are not important for avoiding fire for most of these ground-living species. For large, mobile insects like butterflies and grasshoppers, rockiness can be important for increasing diversity [15], but for different reasons (e.g. improved thermal advantages, food plant availability, shelter from predators), and rock crevices enable protection from fire for grasshoppers elsewhere in Africa [35]. In contrast, our subjects were largely small and cryptic, and found micro-refuges not immediately apparent to the human eye. The little effect on interactions between time-since-fire and rockiness suggest that these assemblages recover no matter what the levels of rockiness. Higher species richness in the 7-year category was not surprising, and is most likely due to the recovery of the structural complexity of the vegetation after 7 years without fire compared to the 3-month and 1-year categories with their less complex vegetation structure.

**Fig 6:**
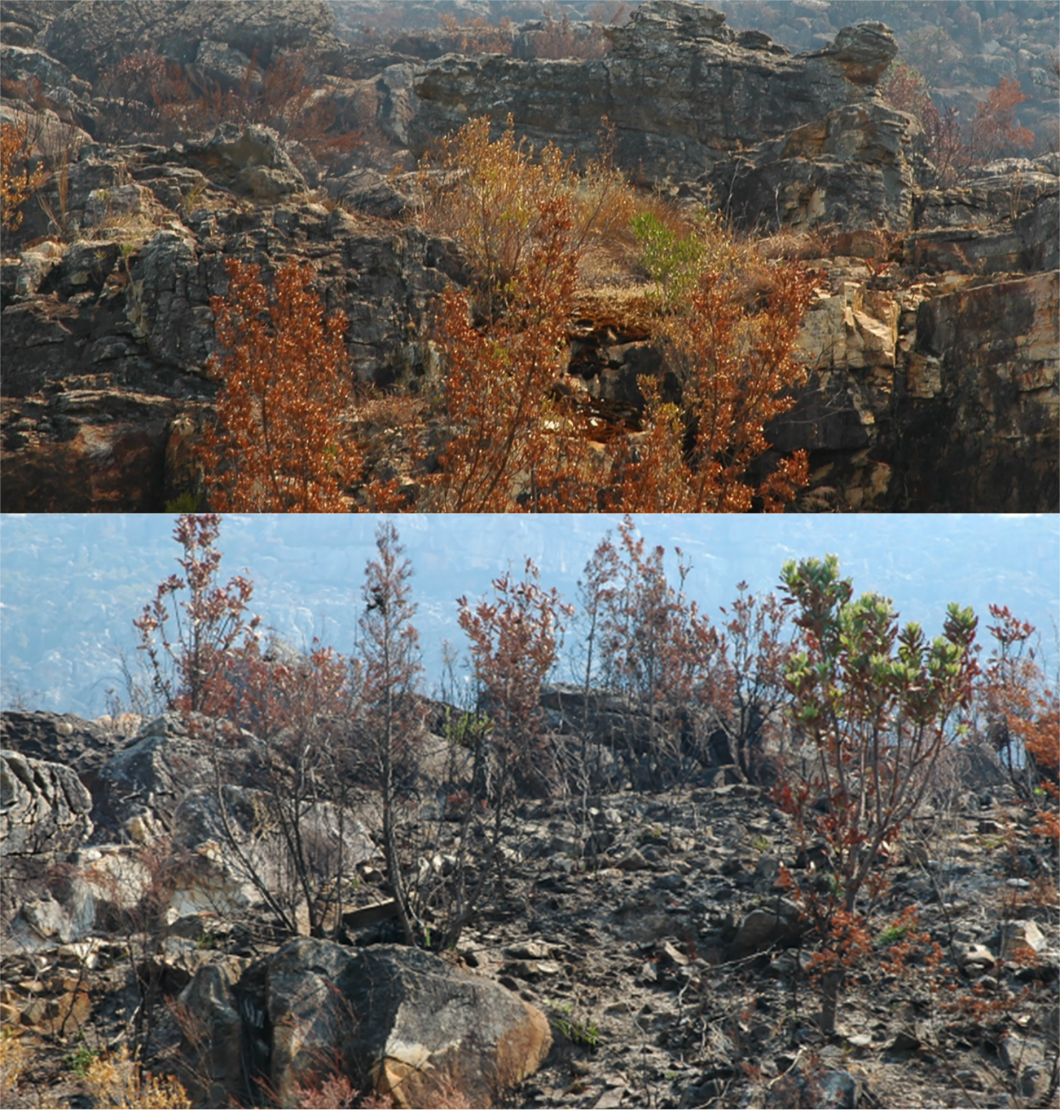
Living plant refuges immediately after a fire has passed.

Here we show that overall arthropods survived fire with similarities in species richness between different time-since-fire categories, supporting previous findings, where ant species richness was not affected by fire in African savanna [34]. However, contrasting results coming from Argentina have shown that unburned forests had greater species richness of beetles [36], and of beetles and ants compared to burned forests [37]. These were attributed to the loss of canopy and increased availability of sunlight [36]. Dissimilarities between these findings and ours may be due to different vegetation types in the two studies, with fynbos dominated mostly by sparse, shrubby vegetation. Furthermore, ants and some beetles (20% of those collected) are predators, which are not dependent on plants for food directly, but on the availability of prey. So ants and predatory beetles could survive in burned habitats provided prey is still available. Ants and beetles may have survived by finding local refuges during the fire. It is common in fynbos for individual plants to escape complete burning, especially at rocky sites (Fig 6). This was the case here, enabling some arthropods to survive and then become a source population. In turn, ants probably returned to their nests below ground, where fire has little effect on them [38].

Although overall arthropod species richness was not influenced by different time-since-fire categories, our results showed variation among taxa, time-since-fire categories did not influence species richness of beetles and ants but affected species richness of cockroaches and mites. This variation emphasizes the importance of focusing on many taxa i.e. the multi-taxon approach [24]. Greater plant biomass accrues over time, both living on the plant and dead on the ground. Leaf litter serves as food and shelter for some arthropods and increases their diversity [39,40]. This is probably why there was higher mite and cockroach species richness in the 7-years since fire category. This was supported by the indicator value (IndVal) analysis identifying the 7-year category as supporting more unique species than the other two time-since-fire categories. Our results are consistent with those from Georgia, where there was a positive correlation between mite density and recovery of plant cover [12].

Heat from fire must kill many individual arthropods [38], although some mites can survive as much as 42°C [41]. However, fire-induced changes to habitat structure can be the major driver of secondary losses of local species, as seen here by the low mite and cockroach species richness. Fire simplifies the habitat, reduces food availability and oviposition sites. This leads to an increase in competition for the limited resources, while also increasing exposure to predators [36,38]. However, arthropod responses to fire depend on ecosystem type and arthropod taxon. While arthropods showed no change in species richness after savanna and pine forest fires [42,43], mites and cockroaches did so in our fynbos system. Yet, we observed similarities in cockroach species richness between the 3-month and 7-year categories, despite the differences in habitat structure. Low cockroach species richness in the 1-year category could be due to secondary losses of species from reduced food and shelter. We expected low species richness of cockroaches in the 3-month and 1-year categories through fire causing a reduction in leaf litter deposition, the primary food source for cockroaches [11,44]. Overall, these results suggest that arthropod responses are complex, depending on species, traits, and micro-features of the landscape, as well as on gross vegetation type and structure.

One year post fire had the greatest abundance of arthropods, which is similar to another study in the fynbos [9]. This was attributed to additional floral resources, as many plants in the fynbos flower within a year of fire [9,17]. Here, it was mainly ants and beetles that were driving this result, with both groups using fynbos flowers as a supplementary resource. Furthermore, both are predominately predatory and this may responding to the increase in pollinators as prey. Higher ant and beetle abundance in the 1-year category compared to the other two, could possibly be due to there being more bare ground [45], making foraging easier for them [46]. Although the 3-months category there is more bare ground, there is also low plant biomass and low herbivorous insects [47], which serve as prey for ants. This may be due to the intermediate disturbance hypothesis, with both the fire species and mature fynbos species both being present, although if this was the case then we would expect to see higher species richness as well [48].

We show that some arthropods are highly resilient to fire, as shown by similarities in beetle, mite and cockroach abundances among all time-since-fire categories. The IndVal analysis supported species resilience to fire, suggesting fire tolerance by a cockroach and two beetle species that were indicators of the 3-month sites, and a mite that was an indicator of the 1-year sites. It seems that these arthropods either find very small refuges in and among the roots and rock/soil crevices to avoid the flames moving by overhead, or it is possible that these beetles recolonized burned sites rapidly after a fire, especially as fire triggers germination of some plant species and the spouting of geophytes [5,11,36].

Fire did not influence species richness and abundance of some arthropod taxa, yet it affected species composition of all the arthropod taxa. As a result, we recommend the use of different diversity measures. Similar findings, where there were differences in species composition but not in richness, have been reported on impact of fire on mites in pine forests [42], and on mites and ants in savanna [29,43]. Similarly to our findings, ant species composition has been reported to be affected by fire in forests in Argentina [37].

Here there was succession following time-since-fire, as shown by the IndVal results. Three species were generalists, commonly occurring in all three time-since-fire categories, while other species were characteristic of a certain time-since-fire category, suggesting great differences among the various species. In South African grassland, fire alters plant species composition and contributes to homogenisation of habitat [49]. However, over time the fynbos changes, with vegetation complexity re-establishing with time-since-fire, from mostly bare ground at 3 months to largely vegetation cover (but still with bare patches) at 7 years, along with associated great changes in micro-structure, -climate and -habitat, and supporting different arthropod assemblages. Then there are traits, including mobility and colonization abilities, of the arthropods themselves. The seven indicator species that were shared by the 1-year and 7-year categories, appear to be colonizers from unburned areas, being largely absent from the 3-month category, especially given that there were no indicator species shared by the 7-year and 3-month categories. In contrast, it was no surprise that the 3-month and 1-year categories shared four ant indicator species, as they can retreat underground and are generalists.

Assemblage turnover with time-since-fire involved both a continuum of generalists, like the four ants above, and then a large set of either recovering populations or re-establishing populations from unburned areas. The complex interaction between species’ traits and features of the habitat are seen when rockiness interacted with time-since-fire, especially for cockroach species richness and abundance. Finally, we must view all these complex interactions in a historical perspective, with fire having been a feature of this landscape for many millennia, and a strong selective force on the species present. We see this among the endemic grasshoppers, with the flightless lentulid *Betiscoides* sp., which are associated with their fire-driven Restionaceae food plants, showing colour morphs in response to fire [50].

In summary, we are seeing the survivors not just of the immediate past fire, but also in terms of evolutionary response. It is the survivors we have recorded here of both a historical and a current highly dynamic fire-driven system. It appears that different arthropod species have been selected to deal with this highly dynamic and unpredictable impact in different ways. While they have been partly constrained by phylogeny, within those constraints, those still around today have found a myriad of ways to survive this trying environment. Hence, monitoring would be best using a suite of taxa with a multiple strategies to overcome fire, this would capture the range of responses.

## Supporting information

S1 Table Species with significant indicator values (IndVal) across time-since-fire categories. 3M (3-month category), 1Y (1-year category), 7Y (7-year category), N (non-rocky sites), R (rocky sites). * p < 0.05, ** p < 0.01, *** p < 0.001.
(DOCX)
