# Supplementary material for: Only multi-taxon studies show the full range of arthropod responses to fire
Source: PLoS One. 2018 Apr 3;13(4):e0195414. doi: 10.1371/journal.pone.0195414 (PMC5882145; doi:10.1371/journal.pone.0195414)
Supplement: S1 Table — 3M (3-month category), 1Y (1-year category), 7Y (7-year category), N (non-rocky sites), R (rocky sites). * p < 0.05, ** p < 0.01, *** p < 0.001. (DOCX) [file pone.0195414.s001.docx]

**Supporting information 1 Table**

|  | **3M-R** | **3M-N** | **1Y-N** | **1Y-R** | **7Y-N** | **7Y-R** |
| --- | --- | --- | --- | --- | --- | --- |
| **3-month category** |  |  |  |  |  |  |
| Tenebrionidae sp. 1 | 0.3** |  |  |  |  |  |
| Coleoptera sp. 1 |  | 0.45* |  |  |  |  |
| Sphaerotheriidae sp. 1 | 0.45* |  |  |  |  |  |
| Blaberidae sp. 1 | 0.56** | 0.56** |  |  |  |  |
| *Pachycondyla* sp. 1 | 0.52** | 0.52** |  |  |  |  |
| **1-year category** |  |  |  |  |  |  |
| *Pachycondyla* sp. 2 |  |  |  | 0.47* |  |  |
| Acari sp. 1 (mite) |  |  | 0.69*** | 0.69*** |  |  |
| Chrysomelidae sp. 1 |  |  | 0.52* | 0.52* |  |  |
| **7-year category** |  |  |  |  |  |  |
| Carabidae sp. 1 |  |  |  |  | 0.71*** |  |
| Carabidae sp. 2 |  |  |  |  | 0.49** |  |
| Sylvanidae sp. 1 |  |  |  |  | 0.49** |  |
| Chrysomelidae sp. 2 |  |  |  |  | 0.52** | 0.52** |
| Cryptophagidae sp. 1 |  |  |  |  | 0.44* |  |
| *Tapinoma* sp. 1 |  |  |  |  | 0.64** |  |
| *Tetramorium* sp. 1 |  |  |  |  | 0.51* |  |
| Blattodea sp. 1 |  |  |  |  | 0.49* |  |
| Blattodea sp. 2 |  |  |  |  | 0.48* |  |
| Blattodea sp. 3 |  |  |  |  | 0.42* |  |
| Blattodea sp. 4 |  |  |  |  |  | 0.47* |
| Polydesmidae sp. 1 |  |  |  |  | 0.43* |  |
| Acari sp. 2 (mite) |  |  |  |  | 0.73*** | 0.73*** |
| Acari sp. 3 (mite) |  |  |  |  | 0.56* | 0.56* |
| **3-month and 1 year categories** | | | | | | |
| *Ocymyrmex* sp. 1 |  | 0.66** | 0.66** | 0.66** |  |  |
| *Pheidole* sp. 1 |  | 0.44* | 0.44* |  |  |  |
| *Pheidole* sp. 2 | 0.87** | 0.87** | 0.87** | 0.87** |  |  |
| *Tetramorium* sp. 2 | 0.63* | 0.63* | 0.63* | 0.63* |  |  |
| **1-year and 7-year categories** | | | | | | |
| Curculionidae sp. 1 |  |  | 0.37* |  | 0.37* |  |
| Staphylinidae sp. 1 |  |  | 0.61** | 0.61** | 0.61** | 0.61** |
| Elateridae sp. 1 |  |  | 0.55* | 0.55* | 0.55* | 0.55* |
| Blattodea sp. 5 |  |  |  | 0.56* | 0.56* | 0.56* |
| Acari sp. 4 (mite) |  |  |  | 0.58* | 0.58* | 0.58* |
| Acari sp. 5 (mite) |  |  |  | 0.53* | 0.53* | 0.53* |
| Acari sp. 6 (mite) |  |  | 0.81*** | 0.81*** | 0.81*** | 0.81*** |
| **All three categories** |  |  |  |  |  |  |
| *Camponotus* sp. 1 |  | 0.59** | 0.59** |  | 0.59** |  |
| *Camponotus* sp. 2 | 0.77*** | 0.77*** | 0.77*** | 0.77*** | 0.77*** |  |
| Harpagophoridae sp. 1 | 0.72*** | 0.72*** | 0.72*** |  | 0.72*** |  |
